# Engaging the patients and prescribers of tomorrow: educational engagement to build school pupil awareness of antimicrobial resistance

**DOI:** 10.1016/j.infpip.2026.100568

**Published:** 2026-07-15

**Authors:** Sam Watkin, Elaine Cloutman-Green, Christopher Little, Emilie Howes, Peter Collins, James Amos

**Affiliations:** a Civil, Environmental and Geomatic Engineering, University College London, London, UK; b Camila Botnar Laboratories, Great Ormond Street Hospital for Children NHS Foundation Trust, London, UK; c Pfizer UK LTD, Tadworth, UK

**Keywords:** Antimicrobial resistance, AMR awareness, Education

## Abstract

**Background:**

Effective public engagement in the challenges posed by antimicrobial resistance (AMR) is crucial to increase awareness and build positive behaviours relating to antimicrobials. Engaging children is essential in this, to shape their future perceptions of AMR and antimicrobial stewardship, and to influence their families and peers on the appropriate usage of antimicrobials. To achieve this, effective educational interventions must be developed, with clear assessments of their efficacy to improve their future development and reach.

**Aim:**

To evaluate the efficacy of an educational event linked to AMR on student and teacher awareness.

**Methods:**

A three-day educational event was performed with 405 student attendees, with ages ranging from 7 to 14 years. The event included a superbug exhibition, facilitator-led sessions, industry-led sessions and healthcare-led sessions. Qualitative opinions were captured using a notice board, and quantitative event effectiveness evaluations were captured both pre- and post-event.

**Results:**

Significant increases in self-described awareness of AMR were observed in both students and teachers following the event. Students had a lower self-assessed knowledge before the event, and exhibited a greater increase in knowledge compared with teachers. Qualitative findings showed themes of active change amongst the student cohort.

**Conclusion:**

The educational event was able to significantly increase the self-described awareness of AMR amongst the participants. Similar event formats should be considered for use when aiming to engage this demographic in the challenges posed by AMR.

## Introduction

Antimicrobial resistance (AMR) is a major threat to public health, with a forecasted 1.91 million deaths directly attributable to AMR in 2050 and has been identified as a “super-wicked” problem, requiring multiple inter-disciplinary strategies to effectively manage it [1,2]. A key method to build wider awareness of AMR is through public engagement strategies. Building public awareness is of crucial importance to address AMR, with public awareness campaigns having been shown to result in a 6.5–23.8% reduction in mean antibiotic usage in Europe over a 10-year period [3]. Of particular importance is building engagement of children with AMR, with early education on the subject helping develop long-term awareness of the risks posed by AMR and understanding of how they can limit their impact on the development of AMR [4].

A significant barrier to effective community AMR stewardship is the perceived need for antimicrobials, for example from parents seeking care for their children, which can lead to pressure on prescribers to provide antibiotics when they may not be necessary [5,6]. Building an awareness in children of AMR and the consequences of antimicrobial over-prescribing may influence parental decision-making, while further shaping their own attitudes to antimicrobial usage. As such, engagement with children in the appropriate usage of antimicrobials is crucial to effectively building long-lasting community awareness and behavioural change linked to AMR.

To effectively engage children in AMR, the selected engagement technique must be appropriate and based on assessment of the target audience, evaluating their existing knowledge, levels of interest and learning style. Further considerations into the cost, longevity and reach of the engagement intervention must also be considered [2]. Events which seek to educate children on AMR using performing arts and community science events have shown efficacy in increasing awareness of AMR [6]. Here, an educational event targeting school children in England was performed, with the effectiveness of the event at improving participant knowledge of AMR assessed using the event organizers' evaluation and feedback questions.

## Methods

A 3-day educational event targeting primary and secondary schools (attendees from Key Stage 2 and Key Stage 3; 7–14 years old) was undertaken during World AMR Awareness Week (WAAW) (18–24 November 2023). The workshop was developed as a component of the Superbugs: Join the Fight! initiative, sponsored by Pfizer UK. Schools were invited to participate through a national campaign which was disseminated by e-mail to schools which had previously engaged in similar sessions during WAAW sponsored by Pfizer UK. The event consisted of a superbug exhibition (showcasing the Superbugs: Join the Fight! initiative), facilitator-led sessions (engage students and schools in Superbugs: Join the Fight! initiative), volunteer-led sessions focusing on careers in the pharmaceutical industry and the relationship with AMR, and healthcare professional-led sessions on the relevance of their work to AMR. The workshop involved a range of activities, including science and career talks, interactive experiments, and demonstrations. Consent for attendance was acquired through normal school permission routes.

Both quantitative and qualitative data were collected by the event organizers, with mixed methods used to evaluate the effectiveness of the event. Qualitative feedback was collected throughout the event on noticeboards where attendees could write free-form comments. Pre- and post-event feedback evaluation forms were provided to attendees to capture quantitative information on the effectiveness of the event on informing them on AMR, with questions focusing on knowledge and awareness of AMR. Scores were collected using a Likert scale, with the questions and scales used presented in Table I.

**Table I:** Comparable questions asked to students and teachers before and after the event on their knowledge of antimicrobial resistance

Likert score	Student	Teacher
	How much do you know about antibiotic resistance?	Before/after attending the schools day, how aware were you about antimicrobial resistance (AMR)?
1	Nothing	Not at all aware
2	A little	Not very aware
3	A lot	Neutral
4	–	Somewhat aware
5	–	Very aware

Qualitative responses were collated and a word cloud of generalized themes mentioned was created using Nvivo 14. Quantitative measures of pre- and post-event knowledge were collected and the mean average score and standard deviations were calculated, with shifts in average Likert score calculated. Statistical significance between scores was calculated using a paired Wilcoxon signed rank test (performed in R v.4.5.0).

## Results

**Figure 1:**
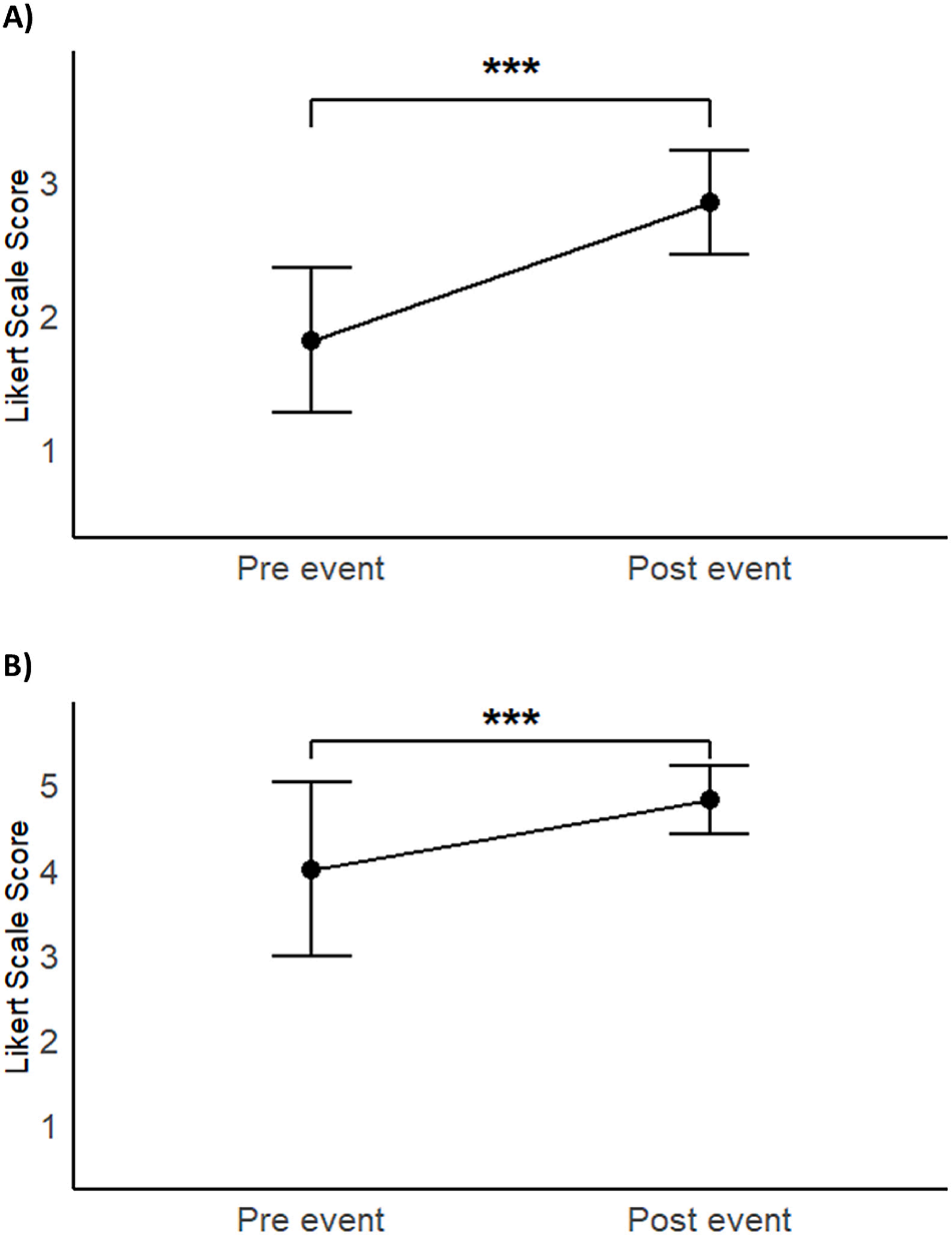
Changes in student and teacher awareness of antimicrobial resistance (AMR) after the event. Changes in mean awareness scores for AMR for (a) students and (b) teachers were measured. Statistical significance was determined using a matched-pair Wilcoxon signed rank test. Error bars show ± standard deviation. ∗∗∗ *P*<0.001, a: *N* = 395; b: *N* = 24.

A total of 405 children attended the event from eight primary and secondary schools, with a 98% response rate to the event evaluation questions. Before the event, students showed a mean average awareness of AMR on the provided 1–3 Likert scale of 1.81 ± 0.54. After the event, an increase in average score of 1.02 was identified, with an average post-event awareness score of 2.83 ± 0.39 (Figure 1a). A similar increase in average awareness score was observed for teachers attending the event, with an increase of 0.81, from 4.00 ± 1.02 to 4.81 ± 0.40 (Figure 1b).

**Figure 2:**
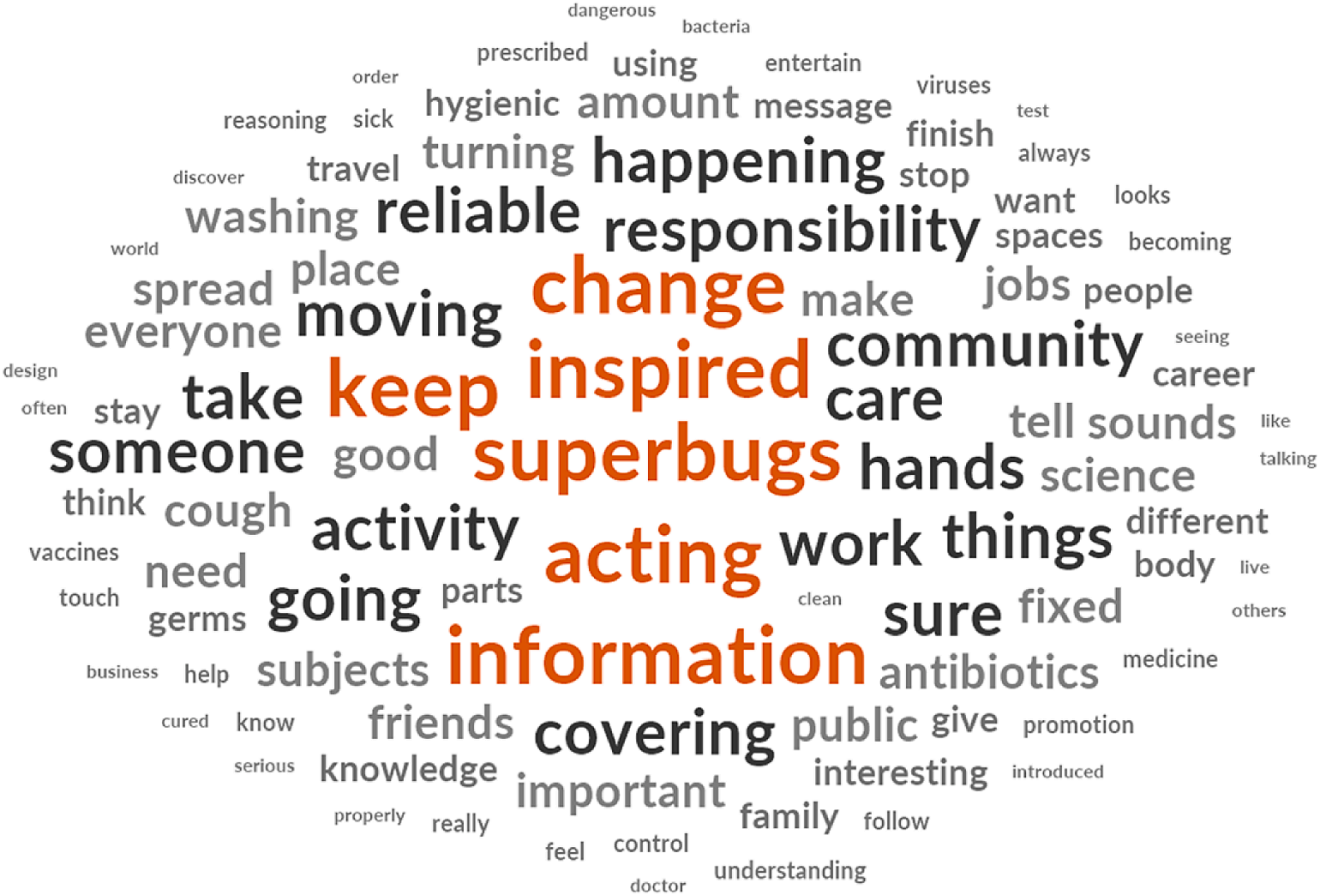
Word cloud of generalizations from free-form student quotes gathered during the event. *N* = 233 responses provided.

Student responses indicated themes of inspiration (shown by frequent uses of “change”, “inspired” and “acting”) during the event. Further frequently mentioned responses included themes of caring and community awareness (Figure 2).

## Discussion

Ensuring effective engagement of children in AMR is essential, both in building immediate understanding of the challenge of AMR and in developing early awareness of the challenge AMR poses in the patients and prescribers of tomorrow. Here, a multi-day educational event was shown to significantly increase student awareness of AMR, with an average final Likert score of 2.83/3 and a difference of 1.02 between pre- and post-event mean average scores. This finding is in line with a similar educational session on groups of school children, with other science events having led to increased awareness of AMR [7]. Currently, a wide range of AMR messaging techniques are utilized to target children. Such measures include social media, hands-on campaigns and performance arts [6]. For such interventions to be successful, they must be appropriately pitched to their intended audience, take into account the likely level of pre-existing knowledge and actively engage with the audience [2]. The findings of this study, in conjunction with other published evidence, illustrate the efficacy of this form of educational intervention in increasing student awareness of AMR, with such interventions a promising avenue for future engagement activities with child cohorts.

In addition to the benefits observed for the student attendees, a significant increase in AMR awareness of teachers was also demonstrated, with a mean final Likert score of 4.81/5, albeit with a small increase of 0.81 from the mean pre- and post-event scores compared with the student cohorts. Multiple AMR awareness strategies are targeted to adult audiences, with national campaigns including the Antibiotic Guardian initiative and public health messaging from the UK Health Security Agency [8,9]. Evidence has shown that an intervention primarily targeted at children has improved adult awareness of AMR, with a similar finding observed here [10]. Improved adult awareness is of significant benefit, influencing expectations from health providers and empowering the delivery of education on AMR to others, propagating this awareness.

In conclusion, the 3-day educational event on AMR was effective at increasing student and teacher awareness on AMR, with key takeaway messages of inspiration and active change evidenced by students. Further examination of the long-term impact of such interventions would support their implementation, helping evidence the role of science engagement events in shaping awareness, attitudes and behaviour after the educational intervention. The results reported here could be further strengthened by incorporating an assessment of long-term behaviour change as well as self-reported awareness. Furthermore, the nature of the study design meant no comparisons with participants who had not participated in the event were possible, which would have further illustrated the impact of the event on AMR awareness. Such educational strategies, however, offer a promising educational intervention to expand community awareness of AMR, helping better address the impact of AMR on public health.

## CRediT authorship contribution statement

**S. Watkin:** Writing – review & editing, Writing – original draft, Visualization, Methodology, Investigation, Formal analysis, Data curation. **E. Cloutman-Green:** Writing – review & editing, Supervision, Project administration, Methodology, Investigation, Formal analysis. **C. Little:** Writing – review & editing, Resources, Project administration, Funding acquisition, Conceptualization. **E. Howes:** Writing – review & editing, Project administration. **P. Collins:** Writing – review & editing, Supervision, Resources, Project administration, Funding acquisition. **J. Amos:** Writing – review & editing, Supervision, Resources, Project administration, Methodology, Funding acquisition, Conceptualization.

## Ethics statement

The manuscript discusses the effectiveness of an education event to improve awareness of AMR as part of a school public engagement programme. Data was anonymous, non-traceable and voluntary, as such ethics was not deemed necessary by the event organisers. Parents of attendees were consented to attend as part of the standard school visitation process, managed by individual educational institutions.

## Funding sources

The scientific engagement workshop was developed as a component of the Superbugs: Join the Fight initiative, sponsored by 10.13039/100009032Pfizer UK.

## Conflicts of interest statement

S.W. and E.C.-G. have no conflicts of interest to declare and were not involved in the design of the education event. C.L., E.H., P.C., J.A. were all employees of Pfizer at the time of the event delivery and were involved in the design of the educational event, but were not involved in the data interpretation components of this manuscript.
